# Kinematics and energetics of the desert locust (*Schistocerca gregaria*) when jumping from compliant surfaces

**DOI:** 10.1242/jeb.248018

**Published:** 2024-12-16

**Authors:** Jessica Taylor, D. Charles Deeming, Gregory P. Sutton

**Affiliations:** School of Life & Environmental Sciences, University of Lincoln, Joseph Banks Laboratories, Green Lane, Lincoln LN6 7DL, UK

**Keywords:** LaMSA, Biomechanics, Energy, Locust, Jumping, Locomotion

## Abstract

Animals often leap from substrates that give way under them, such as leaves, soft ground or flexible branches. This provides an added complexity for latch-mediated spring-actuated (LaMSA) jumping animals because the spring-loaded system often works so quickly that neural feedback cannot adjust for errors caused by a yielding substrate. We studied a LaMSA jumper, the grasshopper, to determine how the mechanical properties of a substrate giving way under them would affect the kinematics of the jump. We measured this by allowing grasshoppers to leap from two diving boards, a long one that could generate a whole range of relative stiffnesses, and a shorter, much lighter, but stiffer board. Substrate stiffness was manipulated by then placing the grasshopper on different locations on that diving board, presenting from 30% of the grasshopper's leg stiffness to 200 times the grasshoppers leg stiffness. For platform stiffnesses that were less than that of the grasshopper, take-off velocity and kinetic energy were reduced, but jump elevation (the jump trajectory) was unaffected. For stiffnesses that were greater than that of the grasshopper, there was no effect on take-off velocity and kinetic energy. When jumping from an extremely light and stiff substrate, recoil of the surface allowed the grasshopper to recover some of the lost energy. Consequently, when jumping from substrates that are less stiff than they are (such as floppy leaves), grasshoppers must contend with lower take-off velocities, but jump direction is unaffected.

## Teaser

**Summary:** When a grasshopper jumps from a springy platform, the physical characteristics of the animal and platform determine the energy efficiency of the jump.

## INTRODUCTION

Insect jumping is actuated by either muscles or springs. For instance, bush-crickets (*Pholidoptera griseoaptera*) use a muscle-actuated system to jump, where the leg muscles contract to extend the legs and this leg extension generates force on the centre of mass (Burrows and Morris, 2003). When extending their legs with a muscle contraction, insects are limited in how much mechanical power they can generate by the force–velocity properties of their muscles (Zajac, 1989; Ilton et al., 2018). By contrast, some insects such as fleas (*Archaeopsyllus erinacei*), froghoppers (*Aphrophora alni*) and grasshoppers (*Schistocerca gregaria*), actuate their jumps by using a spring (Burrows, 2006; Sutton and Burrows, 2011; Bennet-Clark, 1975). These insects initiate a jump by first latching their legs in place and then using large muscles to store energy in a spring-like cuticular structure. After energy is stored in the bending of this cuticular structure, the legs are unlatched and recoil of the spring extends the legs, shooting the animal into the air (Longo et al., 2019). This spring actuation allows the animal to circumvent muscle force–velocity trade-offs and thus generate huge amounts of mechanical power, resulting in very high jump speeds (Ilton et al., 2018). Spring actuated systems using a latch are thus called ‘latch-mediated spring-actuated’ jumpers, or ‘LaMSA’ jumpers for short (Longo et al., 2019).

When initiating a jump, a grasshopper first latches its leg by contracting a muscle within its femur, the flexor tibiae. This locks the legs in place (Heitler, 1977). Once the leg is latched, contraction of the extensor tibiae muscle stores elastic energy by deforming the extensor's apodeme and a thickened band of cuticle at the distal end of the tibia called the ‘semi-lunar process’ (Bennet-Clark, 1975). The grasshopper then relaxes its flexor tibiae muscle, releasing the latch, causing the semi-lunar process and apodeme to recoil. The recoil forcefully extends the metathoracic leg, rapidly converting the stored elastic energy into kinetic energy. The grasshopper's leg extension pushes into the ground below, accelerating the body and allowing it to become airborne (Bennet-Clark, 1975; Heitler, 1977).

Grasshopper kinematics from rigid substrates are well reported in the literature. An adult grasshopper can take off into a jump in an average of 0.025–0.030 s, producing average velocities of 3 to 4 m s^−1^ with average take-off accelerations of ∼60 m s^−2^ (Bennet-Clark, 1975). Grasshopper take-off elevation angles vary between 28 deg, when the grasshopper moves near horizontally, to 104 deg, when the grasshopper moves near vertically and slightly backwards (Sutton and Burrows, 2008); although the average elevation angle is 45 deg (Pond, 1972; Bennet-Clark, 1975). The position of the leg at take-off predicts this elevation (Sutton and Burrows, 2008; Gvirsman et al., 2016). The energetics and power of a jump have also been documented (Bennet-Clark, 1975), with a typical adult male grasshopper producing a mean of 9–11 mJ of energy during a jump. Bennet-Clark (1975) reported maximum mechanical power of 36 mW. By normalizing this power output by muscle mass, the specific peak power output of the spring recoil is between 450 and 2000 W kg^−1^ (Bennet-Clark, 1975). This is much higher than the 100 W kg^−1^ of power that can be generated by direct muscle actuation (Zajac, 1989; Ilton et al., 2018).

Although these well-known kinematics of grasshopper jumps are all from rigid substrates, grasshoppers sometimes jump from compliant substrates, e.g. from grasses, leaves, or small branches. These are substrates from which applied energy from a jumper can be lost to the movement of the substrate. Kinematics and energetics for *S. gregaria* for these situations are not currently reported. In other species, the compliant nature of a substrate affects the kinematics and energetics of movement. For example, Cuban tree frogs (*Osteopilus septentrionalis*, Hylidae, Anura) can recover energy from a recoiling branch (Astley et al., 2015). The orthopteran *Locusta migratoria* has been shown to have great adaptability to different platform compliances. Across a range of substrate stiffnesses, *L. migratoria* is able to optimise its take-off velocities, take-off times and accelerations. Should substrates become sufficiently compliant, however, this optimisation is no longer possible (Mo et al., 2020).

Examining this topic synthetically, a 4 g LaMSA jumping robot, inspired by the grasshopper jumping mechanism, was engineered, constructed and jumped from compliant substrates of varying mechanical properties (Divi et al., 2023). The experiment investigated the relationship between the mechanical properties, specifically the mass and spring stiffness, of the robotic jumper and the compliant platform. The spring stiffness of the robotic jumper (and will be the same definition when mentioned hereafter) is equal to the stiffness of a theoretical spring that would have the same output energy and the same displacement as the robotic jumper. When the robot jumped from a rigid substrate, most of the energy was expended in the jump and only a little is lost elsewhere, for example, as heat (Divi et al., 2023). When jumping from a compliant substrate, however, part of the robot's energy output was lost and contributed to the movement of the platform beneath. The ratio of two key mechanical properties, spring stiffness (*k*, in N m^−1^) and mass (*m*, in kg), of the compliant platform and the robotic jumper, determined the expenditure of the energy output of the jumping robot. For the robotic LaMSA jumper to recover any lost energy from the recoiling platform, the stiffness of the platform had to be greater than the spring stiffness of the jumper and the mass of the platform had to be less than the mass of the jumper. This created the conditions for the platform to recoil preceding the robot losing contact with the platform, and therefore the energy was able to be returned to the robot as it jumped. However, in the opposite stiffness and mass ratio condition, this energy recovery was not observed because the jumper lost physical contact with the platform prior to the platform's recoil (Divi et al., 2023). Whether this principle was applicable to living organisms employing the LaMSA mechanism and jumping from a compliant substrate is unknown.

Our study sought to investigate the effects of a compliant platform on the kinematics of LaMSA jumping in grasshoppers. How energy moves between the grasshopper and the substrate, and how this affects kinematics, such as velocity, elevation, acceleration and time to take-off, have not been previously explored and documented. It was hypothesised that grasshoppers fully, or partially, recover energy lost to a compliant substrate, depending on the stiffness and mass of the animal relative to the platform. Therefore, it was predicted that: (1) when the stiffness of the platform was greater than the stiffness of the grasshoppers' legs (herein referred to as ‘grasshopper stiffness’), and (2) the mass of the platform was less than the mass of the grasshopper, the animal would recover lost energy from the recoiling platform, as observed in the robotic LaMSA jumper (Divi et al., 2023). In the opposite condition, when platform stiffness was less than the grasshopper stiffness, and when platform mass was greater grasshopper, energy would be lost during a jump and thus compromise kinematics.

## MATERIALS AND METHODS

### Subjects

Fifth instar desert locusts of mixed sex (*Schistocerca gregaria*, Acrididae, Orthoptera, Forsskål 1775; hereafter referred to as grasshoppers) were purchased in batches of 10–12 from a local supplier (Lincoln Reptiles Ltd, UK). Grasshoppers were maintained in a plastic enclosure (27×35×16 cm) within an insectary (ambient temperature maintained at 22–23°C). The enclosure contained egg cartons as shelter and grasshoppers were fed *ad libitum* with washed cabbage leaves and had continuous access to water in a gel form (HabiStat H_2_O balls).

Active grasshoppers selected from the colony were weighed using an electronic balance (Sarcotorius analytic Avery Weigh-Tronix) before being transferred in a cylindrical plastic sample pot to the testing room. Grasshoppers were left for 30 min to acclimatise to the new environment (a room temperature of 20–21°C) before data collection began. This research was conducted in compliance with ethical standards.

### Experimental set-up

**Fig. 1.**
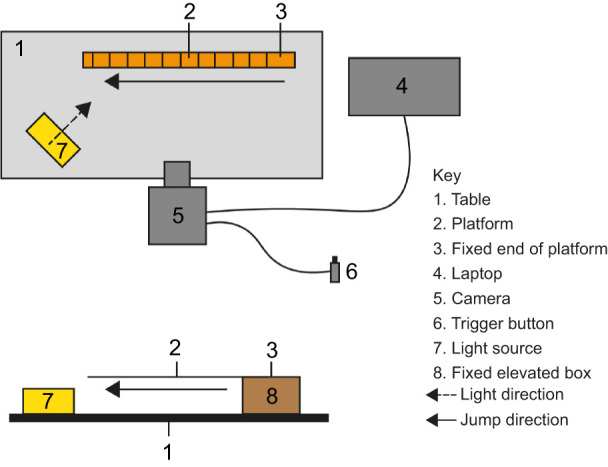
**Experimental set-up for recording of jumps.** (A) Top view. (B) Camera view. The platform (2) was fixed with Blu-tack to the fixed elevated box (8), allowing the platforms to be changed throughout the course of the experiment. For a scaled illustration of the platforms, see Fig. S1.

The recording set-up is illustrated in Fig. 1. Platforms of basswood (*Tilia americana*) of differing sizes (see dimensions defined below), purchased from a local supplier (Hobbycraft, Lincoln, UK), were secured in turn on the top of a rigid wooden box (measuring 13×5×10 cm), that was secured to a table. A 30 cm ruler was fixed to the side of the box to allow for video calibration. All components were secured using re-usable adhesive (Blu-tack^®^, it was ensured that this method of adhesion did not add compliance to the system through checks on the films that the platform did not move at the fixed end). The set up was illuminated using a generic floodlight. A high-speed camera (FASTCAM Mini AX with an aspherical LD XR Di SP Tamron AF 28-75 mm F/2.8 F MACRO 67 A09 lens) was positioned approximately 40 cm from, and manually focused on, the platform prior to each video recording. The camera was connected to a laptop computer (Dell G3 15 P75F) used to record the jumps using Photron FASTCAM Viewer recording software (v. 3.6.9.1) set to continuous recording at 1000 frames s^−1^ and a shutter speed for one frame was 1/10,000 s. The trigger mode set to ‘centre’, which meant that the recording system saved the video footage for 3 s pre- and post- pressing the trigger.

For control jumps, a basswood platform (0.8×5×61 cm, depth×width×length) was secured to the top of the wooden box, where the fixed portion served as a rigid platform. The two experimental conditions (A and B) used basswood boards that were compliant to varying degrees and were secured to the wooden box in a way so that the compliant portion of the platform could overhang, like a sprung diving board over a pool. The compliant platforms were crafted from pieces of basswood 0.2 cm in thickness and were designed to vary in stiffness and mass, relative to the grasshoppers' stiffness and mass. From here onwards, the relationships between the platform and grasshopper are referred to in relation to the mass ratio (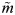) and stiffness ratio (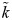), being less than, or greater than, 1.0 (where the stiffness and mass of the grasshopper is the numerator, and the stiffness and mass of the platform is the denominator, see Eqns S1 and S2 in the Supplementary Materials and Methods). Platform A measured 2.5 cm wide×61 cm long, mass 6.34 g and secured to the end of a box using re-usable adhesive. Platform B was 2.5 cm in width and only 12.4 cm in length with the end portion of this platform, measuring 2 cm, fixed to the supporting box, and at 1.01 g weighed less than the grasshoppers, which averaged 1.13±0.04 g.

The lengths of platforms A and B were divided up into equal parts by lines set 5 cm apart (measurements began from the distal end of the fixed portion of the platform, see Fig. S1). To contextualise this, the mean grasshopper body length was 3.49±0.04 cm, making the lines 1.43 times longer than the average body length. Platform stiffnesses were manually measured at each of the 5 cm increments by measuring the distance of platform displacement under a calibrated weight of either 1, 2, 10, 20, 50 or 100 g (see Eqn S3 in Supplementary Materials and Methods), prior to filming the jumping. From here onwards, these marked positions on all compliant platforms will be referred to as ‘line(s)’, where line 1 was the stiffest position on the platform and line 10 was the least stiff and 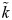 varied along the length of the platforms (see Table S1). Differences between the two experimental groups were in the properties of the platform, the number of jumping positions along the platform and the number of jumps per grasshopper.

### Experimental procedure

For control jumps, grasshoppers were placed on the rigid basswood surface and encouraged to jump from right to left by tapping on nearby surfaces to create a sudden sound or by using a small paintbrush to stroke the abdomen. Active, jumping grasshoppers were selected from the enclosure, but if grasshoppers behaved abnormally during the jumps (e.g. jumped using only one metathoracic leg) they were excluded from the analysis. Using vernier callipers (Starrett), each grasshopper's tibia length was measured to an accuracy of 0.1 mm after experimental data collection and before being returned to the enclosure.

Three control jumps were recorded from each grasshopper, with 2 min between jumps to allow for rest. Grasshoppers are able to repeatedly jump without measurable fatigue (up to 61 consecutive jumps) (Goode and Sutton, 2023), although a 2 min rest was implemented for the additional use of allowing time to process the films between jumps. The sample size of grasshoppers and total number of jumps used for control jump analysis are shown in Table S1. After control jumps were recorded, grasshoppers were jumped from each line along one of the two platforms, totalling 10 experimental jumps from each individual for platform A and two experimental jumps for the shorter platform B with 2 min rests between each jump. Each grasshopper jumped from each line in a random order and were jumped sequentially allowing for a 2 min recovery period between jumps. The grasshopper was placed on the platform so that their centre of mass was between the marked line and 2 cm behind the line for each jump.

**Fig. 2.**
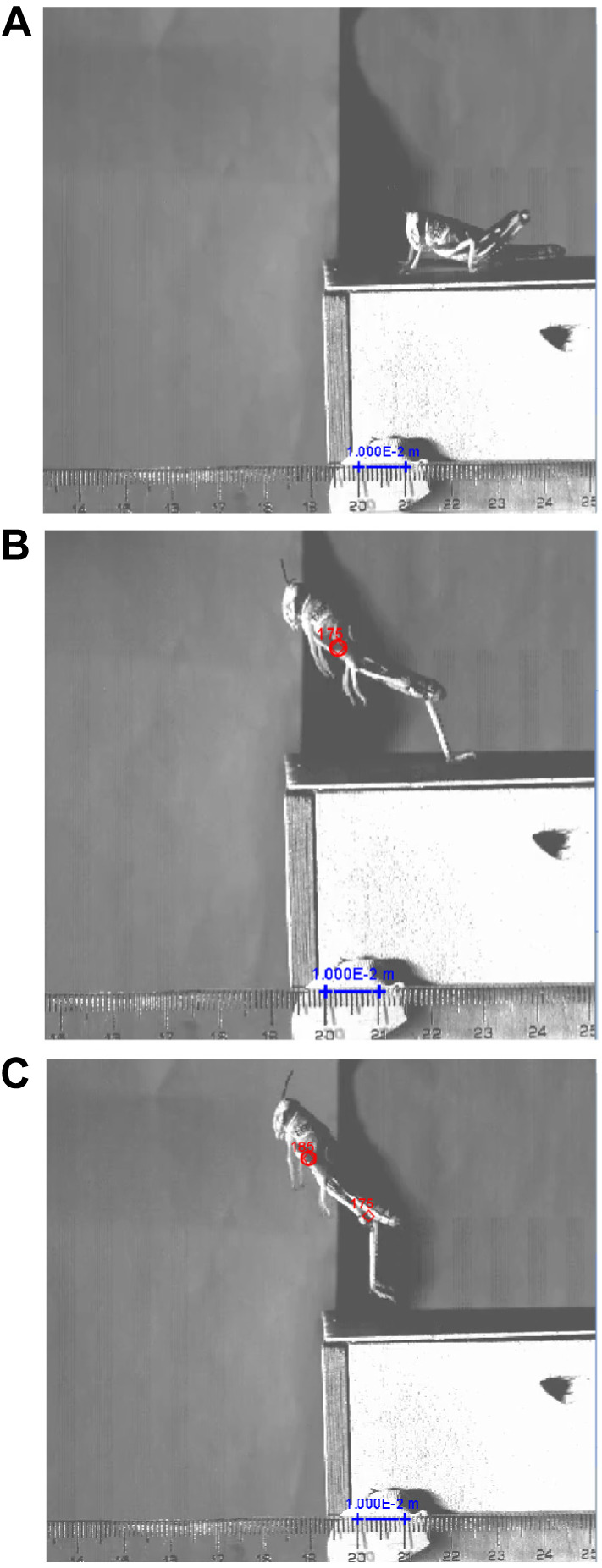
**Example still images from a high-speed video of a control grasshopper jump.** (A) Example of a control grasshopper pre-jump with a calibration to 1 cm (blue bar in A) of film. Close-up images from a grasshopper control jump (B) at the last frame of tarsi–platform contact (0 ms) and (C) 10 frames later (10 ms), displaying the plot points at the same coxa–body joint at 0 and 10 ms.

Videos were digitized using Tracker Video Analysis and Modelling Tool (v. 6.0.3, Open-Source Physics, 2022). Each video was calibrated to scale using the 30 cm ruler in camera view (Fig. 2A). Initial measurements were made by tracking the metathoracic leg joint or on the caudal–ventral extremity end of the pronotum, depending on which area was continuously visible throughout the footage. Positions were measured at the last frame of tarsi-platform contact and 10 ms later (see Fig. 2B,C).

For experimental jumps only, a second set of plots were placed in the software beneath the left tarsus of the grasshopper in the first frame of visible metathoracic movement and the second being the last frame of tarsi-platform contact. These plot coordinates were used for the measurement of platform displacement (m) during the grasshopper jump. An in-software protractor allowed measurement of the angle of metathoracic leg extension for control jumps to calculate the grasshopper's acceleration distance (Eqn S4) and stiffness (Eqn S5 in Supplementary Materials and Methods).

### Analysis

The stiffness of grasshoppers during a jump (*k*_g_) was calculated by determining the stiffness of the linear spring that would produce the same kinetic energy as that observed during a jump from a stiff substrate (the control jumps for each platform). This was done by applying conservation of energy:
(1)

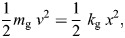
where *m*_g_ is the mass of the grasshopper, *v* is the take-off velocity and *x* is the distance the grasshopper accelerates before take-off; *x* was calculated by determining the distance over which the centre of mass accelerated during the extension of the legs. Gravity has not been considered in the energy calculations because only 1–2% of the grasshopper energy budget is spent on gravity at these masses and distances (Scholz et al., 2006). At the beginning of the jump, the legs were fully flexed (with the distal end of the tibia and the proximal end of the femur almost touching). At take-off, the legs were fully extended, allowing the acceleration distance (*x*) to be calculated in terms of the length of the femur (*L*_f_) and the tibia (*L*_t_) with the law of cosines:
(2)

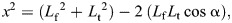
where α is the angle of the femur-tibia joint at take-off, and *L*_f_ and *L*_t_ mark the sides of an isosceles triangle (grasshopper femora and tibiae are similar lengths; Bennet-Clark, 1975). Eqn 2 was then be substituted into Eqn[Supplementary-material sup1]1 to evaluate the effective stiffness (*k*_g_) of the grasshopper legs during a jump.

High-speed movies were used to calculate the following variables using equations detailed in the Supplementary Materials and Methods (Table S2): time to take-off (Eqn S6), velocity (Eqn S7), elevation relative to the deformed platform plane (Eqn S8), platform displacement angle (Eqn S9), acceleration distance (Eqn S4), acceleration (Eqn S10), kinetic energy (Eqn S11), kinetic energy density (Eqn S12), power (Eqn S13), power density (Eqn S14), grasshopper stiffness (Eqn S5), platform stiffness (Eqn S3), stiffness ratio between the platform and the grasshopper (Eqn S1), and mass ratio between the platform and the grasshopper (Eqn S2). Prior to carrying out statistical analysis, a mean of each variable for each grasshopper's set of control jumps was calculated [(variable)_c_]. This was used to normalize experimental values relative to the mean control value of each variable. For all variables proportions relative to the mean of the control was calculated (Eqn S15), except for elevation where difference was used (Eqn S16). Difference was used only for elevation because this variable is a polar quantity and therefore a scalar statistic could not be used.

### Statistical analysis

For control jumps, statistical analysis used R packages lme4 (CRAN.R-project.org/package=lme4; Bates et al., 2015) and lmertest (CRAN.R-project.org/package=lmerTest; Kuznetsova et al., 2017) to perform linear mixed-effects models in R (R-project.org). For experimental jumps, the mean *k*_g_ was 3.42 N m^−1^, whereas *k*_p_ varied according to the position of the line and the platform (see Table S1). The data were split into two categories based on stiffnesses of the platform relative to that of the grasshopper: i.e. *k*_p_<*k*_g_ or *k*_p_>*k*_g_.

Models tested either the relationship between platform stiffness as a covariate and the different variables for when stiffness of the platform was less than that of the grasshopper, i.e. *k*_p_<*k*_g_. When stiffness of the platform was greater than that of the grasshopper, i.e. *k*_p_>*k*_g_, the models tested the effect of platform stiffness as a covariate, but also included platform type as a fixed factor, for the different variables. These latter models initially included an interaction term, which if it proved non-significant, was removed and the model was re-run. All models included a code for each grasshopper as a random factor to control for repeated sampling.

A one-sample *t*-test (Bailey, 1981) was used to test whether mean for each variable at each given platform stiffness was significantly different from a mean of 1.0. The exception was elevation, which was tested against a mean of zero because comparison of the control and experimental jumps was a mean difference rather than a mean proportion. All datasets were tested for a normal distribution using a Shapiro test. All but six datasets were normally distributed, and when tested using a Wilcoxon test, produced the same result as the *t*-test.

## RESULTS

### Effective stiffness of grasshoppers during jumps

The 40 grasshoppers used for this study had a mass of 1.13±0.04 g (mean±s.e.m.) and had jumps from a fixed substrate that took off with a velocity of 1.44±0.03 m s^−1^. The kinetic energy density was 24.79±0.93 J kg^−1^. The length of the tibiae was 15.6±0.187 mm and angle of extension of the femur/tibia joint at take-off was 113±2 deg. The grasshoppers jumped as if propelled by a spring of stiffness (*k*_g_) of 3.39±0.11 N m^−1^ (see Eqns 1 and 2 for how grasshopper stiffness was calculated). There were no significant differences for the different variables between the control jumps for the two groups of grasshoppers used for the two platforms (Table S3).

### Kinematics of jumps when platform stiffness is less than mean grasshopper stiffness

This analysis was based on data collected from platform A only, as this condition occurred when the stiffness of the platform (*k*_p_) was less than the mean stiffness of the grasshoppers (*k*_g_). Time to take-off was significantly positively affected by platform stiffness (Table 1; Fig. S2A). By contrast, elevation was unaffected by platform stiffness (Table 1; Fig. S2B).

**Table 1.**
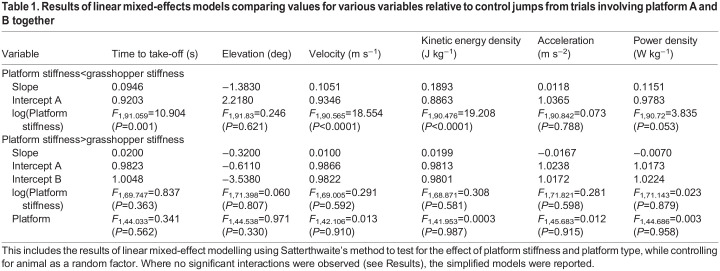
Results of linear mixed-effects models comparing values for various variables relative to control jumps from trials involving platform A and B together

**Fig. 3.**
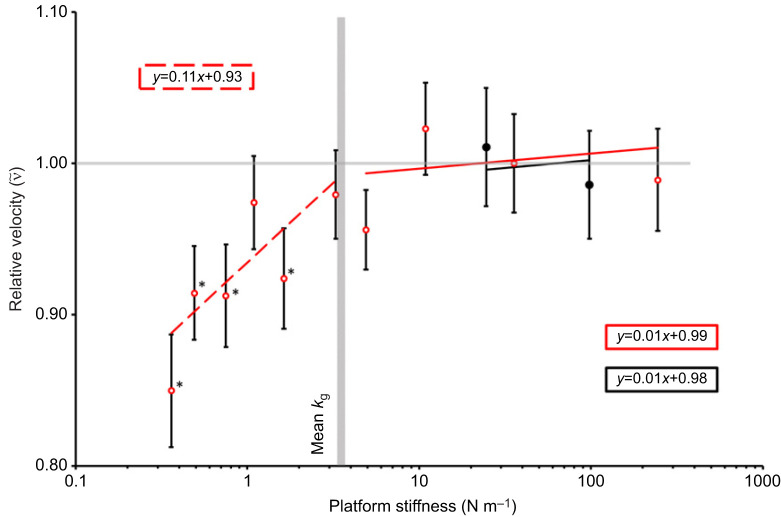
**Relative velocity across different platforms and platform stiffnesses.** The horizontal grey line marks 1, where below this line shows velocity decrease compared with the control jumps. The vertical grey bar shows the mean grasshopper stiffness (3.42 N m^−1^). Asterisks indicates data points significantly differing from 1.0 (where *v*_g_=mean *v*_c_). Trendlines were generated based on output from linear mixed effect regression analysis of the data (see Table 1). Values are means±s.e.m. of *n*=40 [*n*=21 for platform A (red) and *n*=19 for platform B (black)].

**Fig. 4.**
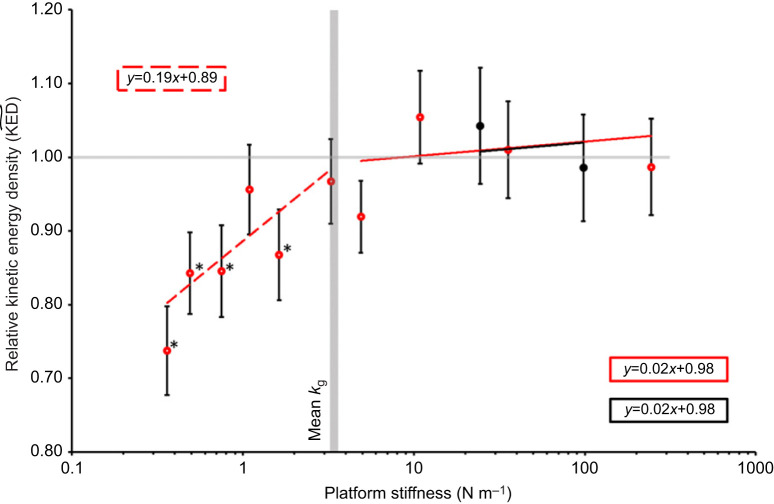
**Relative kinetic energy density across different platforms and platform stiffnesses.** The horizontal grey line marks 1, where below this line shows a decrease in kinetic energy density compared with the control jumps. The vertical grey bar shows the mean grasshopper stiffness (3.42 N m^−1^). Asterisks indicate data points significantly differing from 1.0 (where KED_g_=mean KED_c_). Trendlines were generated based on output from linear mixed effect regression analysis of the data (see Table 1). Values are means±s.e.m. of *n*=40 [*n*=21 for platform A (red) and *n*=19 for platform B (black)].

**Fig. 5.**
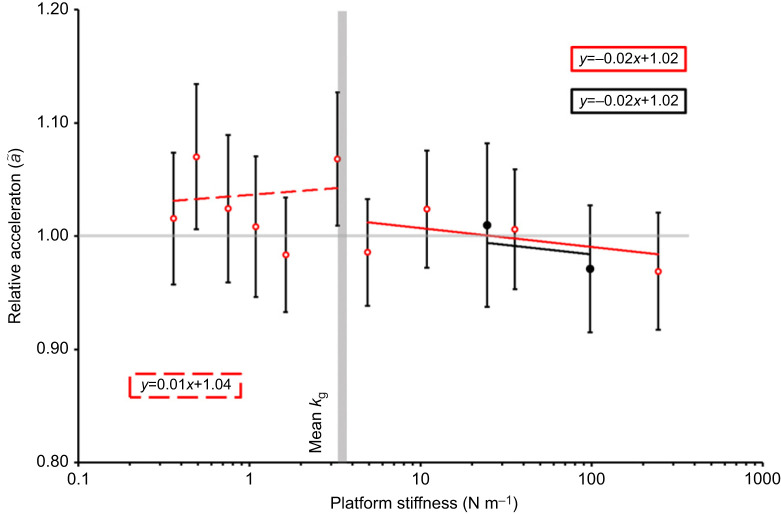
**Relative acceleration across different platforms and platform stiffnesses.** The horizontal grey line marks 1, where below this line shows acceleration decrease compared with the control jumps. The vertical grey bar shows the mean grasshopper stiffness (3.42 N m^−1^). Trendlines were generated based on output from linear mixed effect regression analysis of the data (see Table 1). Values are means±s.e.m. of *n*=40 [*n*=21 for platform A (red) and *n*=19 for platform B (black)].

**Fig. 6.**
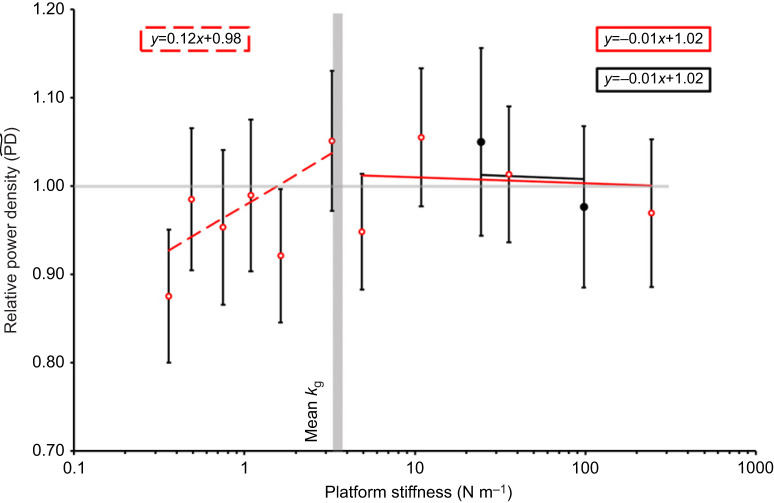
**Relative power density across different platforms and platform stiffnesses.** The horizontal grey line marks 1, where below this line shows a decrease in power density compared with the control jumps. The vertical grey bar shows the mean grasshopper stiffness (3.42 N m^−1^). Trendlines were generated based on output from linear mixed effect regression analysis of the data (see Table 1). Values are means±s.e.m. of *n*=40 [*n*=21 for platform A (red) and *n*=19 for platform B (black)].

There was a significant positive relationship between *k*_p_ and relative velocity (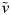) (Table 1, Fig. 3). Likewise, mean values for kinetic energy density relative to control KED (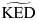) exhibited a strong positive relationship between *k*_p_ and 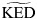, but only until *k*_p_ reached the mean *k*_g_ (Fig. 4, Table 1). Although there was a slight positive relationship between *k*_p_ and relative acceleration 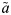 (Fig. 5) this was non-significant (Table 1). When *k*_p_<*k*_g_, like the pattern for acceleration, the relative power density increased with platform stiffness (Fig. 6) but this relationship only approached significance at 0.05 (Table 1). Plots for the relationships between platform stiffness and kinetic energy and power are shown in Fig. S2C,D alongside the equivalent analysis (Table S4).

### Kinematics of jumps when platform stiffness is greater than mean grasshopper stiffness

There was no significant interaction between time to take-off and platform type (*F*_1,78.38_=0.49, *P*=0.486; Fig. S4). In the simplified model, neither platform stiffness nor type significantly affected time to take-off (Table 1). Similarly, elevation was unaffected by the interaction between platform stiffness and type (*F*_1,78.67_=0.88, *P*=0.352; Fig. S2B) and neither the covariate nor fixed factor were significant in the simplified model (Table 1).

There was no obvious effect of platform stiffness or platform type on take-off velocity (Fig. 3). Linear mixed effect regression modelling showed no significant interaction between *k*_p_ and platform type for relative velocity (*F*_2,120.72_=0.92, *P*=0.402). In the simplified model there was no significant effect of *k*_p_ on 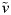
(Table 1). Mean values for kinetic energy density relative to 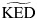 were not significantly affected by platform stiffness and the trendlines had intercepts that were close to the value of 1.0 (Fig. 4). Linear mixed effects regression modelling found no interaction was found between *k*_p_ and platform type (*F*_2,112.30_=0.618, *P*=0.541). There was no significant effect of platform stiffness or platform type on 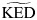 in the simplified model (Table 1).

For relative acceleration values for all platforms exhibited slight negative relationships with *k*_p_ (Fig. 5). Linear mixed effects analysis showed no interaction between *k*_p_ and platform type on relative acceleration (*F*_2,123.81_=0.671, *P*=0.512) and neither platform type nor stiffness showed any significant relationship in the simplified model (Table 1). Mean values for relative power density exhibited slight negative relationships between 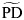 and *k*_p_ for both platforms but were not significantly below 1.0 for either platform A or B (Fig. 6). Linear mixed effects regression modelling showed no significant interaction between platform type and *k*_p_ (*F*_2,122.96_=0.796, *P*=0.454) and the simplified model showed no effect of *k*_p_ or platform on 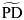 (Table 1).

## DISCUSSION

**Fig. 7.**
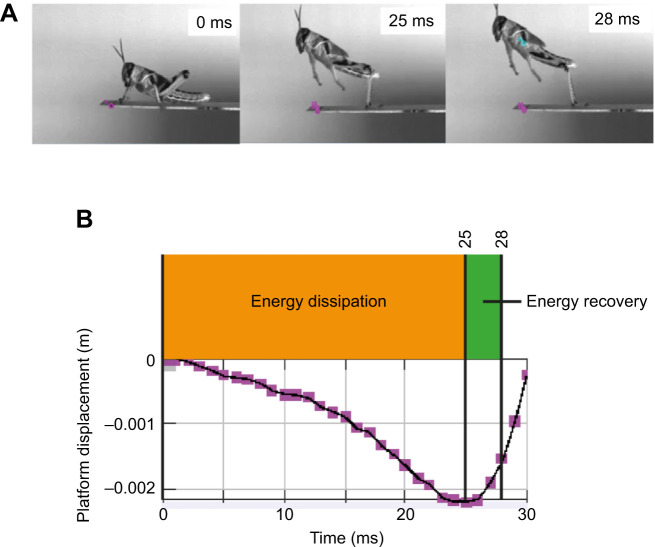
**Energy recovery from a compliant platform.** (A) A grasshopper jumping from a compliant substrate of 24 N m^−1^, platform B. 0 ms was prior to any metathoracic leg extension, 25 ms was at maximum platform displacement (i.e. displaced to its lowest point) and 28 ms was at take-off. The purple marker was a fixed point on the platform that was tracked across the jump. (B) A graphical representation of the displacement of the purple tracked point on the platform. Between 0 and 25 ms, energy is dissipated to the platform as it is moved in a downward direction. Between 25 and 28 ms, the platform moved in an upward direction while the grasshopper remained in contact with it, allowing the grasshopper to recover energy from the recoiling platform during this 3 ms period.

The importance of the platform stiffness on grasshopper jumping kinematics has been highlighted by the present study. We have found here that grasshopper jump kinematics were affected by a substrate only when the substrate stiffness was less than the stiffness of the grasshopper leg. If the stiffness of the substrate was greater than the stiffness of the leg, then grasshopper kinematics were unaffected. The effect of stiffness was observed on the large platform, but this effect could be ameliorated by platform mass (Divi et al., 2023), such that if a platform were heavy enough, then even very low platform stiffnesses would not affect the jump. Only having two mass conditions, we do not have a large enough range of platform masses available to determine the quantitative effect of mass. As pointed out by Divi et al. (2023), for a sufficiently large substrate mass, the stiffness of both the jumper and the substrate become unimportant as the governing mechanics of the jump become a simple exchange of momentum (*m*_p_×*v*_p_=*m*_g_*×v*_g_). If the platform is sufficiently compliant and light enough (as the platforms in this experiment were) then take-off velocities, kinetic energy density and power density decrease as platform stiffness decreases. If a substrate is sufficiently light and stiff, however, energy lost to the substrate can be recovered by the grasshopper, as the substrate recoils (Fig. 7).

### Potential confounding variables

Grasshoppers were repeatedly jumped and although they were given time to recover, there was a possibility for muscle fatigue to occur which could compromise jump performance. Bennet-Clark (1975) showed that grasshoppers were fatigued after 5 to 10 jumps, and jump velocity decreased dramatically within the first 5 min of jumping for second, fourth, sixth instars and adults when repeatedly jumped (Kirkton and Harrison, 2006). Whilst fatigue could have been a confounding variable in this study, the control jumps were filmed prior to the experimental jumps, which ensured that each individual had an optimum jump recorded as a baseline. The experimental jumps were also recorded in a randomised order of stiffness, with each grasshopper having rest time between jumps. Moreover, if fatigue was an issue, a decrease in velocity would be observed for the stiff substrate as well. Although muscle fatigue from repeated jumping would decrease velocity and energy, both variables were maintained under high 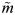 and low 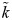 conditions. Moreover, Goode and Sutton (2023) demonstrated that grasshoppers were able to jump over 60 times without measurable fatigue.

Resonance frequency of the platform and the jumper have previously been reported in similar studies, such as Divi et al. (2023), who reported that a faster resonance frequency of the platform, relative to the jumper, being key to measuring energy recovery. There remains much to explore about relative frequency, although we decided to not include this in our study because of the large amount of data focusing on kinematics already being reported. Further investigation is necessary to extrapolate exactly how much energy is being recovered in biological LaMSA systems while keeping resonance frequency in mind.

### Performance of grasshoppers in control jumps

The kinematics of grasshoppers jumping from rigid platforms reported here was comparable to data reported in the literature. For instance, adult grasshoppers jumped at 3.2 m s^−1^ (Bennet-Clark, 1975), which was faster than earlier ontogenetic stages (Gabriel, 1985a). In this study, fifth instar grasshoppers jumped at a mean velocity of 1.45 m s^−1^, which was slower than an average take-off velocity of 1.5 m s^−1^ for fourth instar juvenile grasshoppers reported by Katz and Gosline (1993). These differences may reflect increases in cross section area of the femur which becomes wider through moults to accommodate the increase in the mass of the extensor tibiae muscles (Gabriel, 1985a,b; Bennet-Clark et al., 1990).

Jump elevation is independently controlled by the rotation at the coxae, prior to tibia extension (Sutton and Burrows, 2008). In the present study, the insect jumped with a mean elevation angle of 43.5 deg (Table S3), which was comparable to adult grasshoppers that consistently jumped at 45 deg (Pond, 1972; Bennet-Clark, 1975). Grasshoppers can jump at elevations ranging from 28 to 104 deg (Sutton and Burrows, 2008) but, despite this, the fifth instars jumped here were very consistent. Visual input and a ‘peering’ behaviour prior to the jump allow the grasshopper to jump accurate distances (Wallace, 1959) and jump towards a specific target that requires a precise jump elevation (Sobel, 1990). The combination of a consistent jumping environment, and the independent control of the jump elevation by rotation at the coxae was likely to have created this reliable mean elevation of 43 deg. However, jump elevations in the present study ranged from 19 to 69 deg, which may reflect different jump types (Sutton and Burrows, 2008). For instance, ‘directed’ jumps are preceded by the peering or scanning behaviour, but this is not often observed during ‘escape’ jumps (Sobel, 1990). Though not systematically recorded in this study, this peering behaviour was occasionally observed prior to jumping by some individuals. Grasshoppers that were motivated to jump may be expressing an escape jump that lacked the peering or scanning behaviour (Sobel, 1990) and rotation of the coxae (Sutton and Burrows, 2008) and so may explain a wide range of observed elevations in this study.

### Jumping performance from compliant platforms

Literature around locomotion from compliant platforms is limited and none are available for *S. gregaria*. Orangutans (*Pongo abelii*) can maximise their energy budget from the oscillating movements of branches while swinging from tree to tree (Thorpe et al., 2007). When jumping Cuban tree frogs recover energy from compliant branches, even from a static position, velocity, energy and power output of the frogs are unaffected (Astley et al., 2015). This is achieved by delaying the extension of the hind leg, which increased the time for which the frog is in contact with the substrate (Reynaga et al., 2019). By contrast, compliant perches significantly decrease jump velocity in green anole lizards (*Anolis carolinensis*; Gilman et al., 2012). Mo et al. (2020) found that *Locusta migratoria* were able to jump optimally on complaint ground in relation to their time to take-off, velocity and acceleration.

Grasshoppers usually locomote across compliant vegetation where the ability to recover energy when jumping could be advantageous. It is possible that grasshoppers select where they jump from to optimise energy recovery, therefore enhancing jump performance in the field. This would require perception of mass and stiffness of their environment, and the cognitive ability to perceive the environment. In addition, one recent study has revealed that grasshoppers can filter out some sensory information from visual input, supporting the idea that they have a developed cognitive ability to make informed choices about their environment (Bleichman et al., 2023).

Interestingly, a model robot, inspired by the grasshopper's LaMSA system, can recover energy from a recoiling platform under specific mechanical conditions (Divi et al., 2023). When the platform stiffness was greater than the robot stiffness, energy recovery was observed. When replicating this methodology for the investigation into grasshopper LaMSA jumping, there was a stress on the importance of relationship between the mass and stiffness of the platform and grasshopper leg. Across all ratios of masses and stiffnesses between the grasshopper and the platform, there was no significant influence of the platforms on time to take-off and elevation but there were significant relationships for velocity and kinetic energy density but non-significant relationships for acceleration and power density as proportions of the control values.

### What is not affected by platform compliance?

Adult grasshoppers take around 0.02–0.03 s to take-off, whereas fifth instars can take up to 0.65 s from a rigid substrate (Katz and Gosline, 1993). Experimental times to take off were not greatly different to the control times to take-off and were unaffected by platform mass or platform stiffness. Astley et al. (2015) also found that jump duration by Cuban tree frogs did not vary across differing perch stiffnesses and suggested that the frogs have adapted a robust jumping strategy by maintaining their time to take-off while jumping from various perches in the field. This explanation could apply to grasshopper jumping because to produce the same velocities over a slower time to take-off would create lower accelerations and lower power outputs and would therefore be advantageous to keep a constant time to take-off to continue producing high accelerations.

The differences in elevation compared with the controls were non-significant, suggesting that grasshoppers were able to adapt to maintain their elevations across different compliant substrates of differing masses and stiffnesses. Grasshoppers can maintain precise elevations while jumping, through peering and scanning behaviours (Sobel, 1990) and rotation at the coxae (Sutton and Burrows, 2008), which is supported by the precise elevations from the fifth instar control jumps. Consistent jump elevation also suggests that, no matter the reaction of the substrate, the direction of the force vector of the jump stays constant, allowing recovered energy to apply force in the direction of the jump, instead of knocking a grasshopper off target.

### Connections between velocity and kinetic energy density

The patterns of the relationships between platform stiffness and velocity, kinetic energy and kinetic energy density were similar because they were all calculated based on a similar set of kinematic measurements. Velocity is required to calculate kinetic energy and so changes to velocity were squared in the calculation of kinetic energy. By contrast, changes in mass of the grasshopper would affect kinetic energy in a linear fashion (Eqns S2 and S7), although the mass of each grasshopper was constant during this experiment, and therefore did not affect the kinetic energy budget. A reduction in the grasshopper's energy budget only occurred when the platform had a stiffness less than that of the grasshopper leg. Divi et al. (2023) also found that the platform from which the LaMSA robot jumped from could sometimes recoil before the jumper lost contact with it. While still in physical contact with the platform, the jumping robot was able to recover up to 12 mJ of this lost kinetic energy back into the jump (Divi et al., 2023). This was also observed in grasshoppers because when jumped from a platform with low mass and a high stiffness relative to the grasshopper mass and stiffness (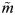
>1 and 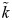
<1), the grasshopper maintained its kinetic energy without dissipation to the platform, which resulted in a maintained velocity relative to the control jumps. Energy recovery was possible from a light and stiff platform, relative to the jumper, because it can recoil fast enough for the jumper to recover energy from it. This was observed in stills of the high-speed films of grasshoppers jumping (Fig. 7).

Grasshoppers were able to recover energy from a platform of high mass and high stiffness, rather than exclusively from low masses as seen in robotic LaMSA systems (Divi et al., 2023). Here, the platform does not necessarily need to be a lower mass than the jumper for energy recovery to occur in biological LaMSA systems. However, there is a point in which the platform became too massive for it to recoil quickly enough for energy recovery to occur. Energy recovery has been demonstrated in grasshopper jumping if 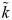
<1, and 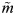
>0.17. This suggests that grasshoppers, and possibly other biological LaMSA jumpers, are more robustly adapted to jump from a wider range of substrate masses than initially hypothesized. However, it is imperative that the stiffness of the platform is greater than that of the grasshopper for it to recoil quickly enough. Platform recoiling was also observable in the high-speed film stills of jumps from the smallest platform (an 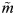
>1 and 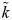
<1 condition), but energy recovery from a platform where 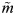
<1 and 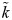
<1 condition (i.e. the stiff end of platform B) could not be observed from the film stills.

For kinetic energy density and velocity to be maintained in nature, the grasshopper would need to select leaves and grasses that are stiffer than their leg stiffness. However, the substrate does not necessarily need to be lighter than the grasshopper, because energy recovery had been demonstrated from platforms that are of greater mass than the grasshopper. Although these conditions are what we could define as optimum jumping conditions, it is unknown whether grasshoppers have the decision-making ability to select for these conditions.

### Connections between acceleration and power density

Acceleration is equal to the velocity at take-off over time, which is required for calculation of power, and power density is power relative to the mass of the grasshoppers' jumping muscles. Consequently, acceleration, power and power density all followed similar trends relative to platform stiffness.

Even though Divi et al. (2023) did not discuss acceleration and power density of the LaMSA robot, it can be hypothesized that these variables would be lower than the control values when the platform mass was high because of the relationship of these variables with velocity and kinetic energy. Data supported this because acceleration and power density of jumps from the smallest platform were similar to the control accelerations, and 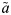 and 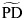 remained ∼1.0 across all platform stiffnesses but were much lower on platform A where the mass was much greater than the grasshopper.

Similarly to velocity and kinetic energy density, acceleration and power density under the 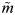 conditions on platform A, were close to 1.0, explaining the maintenance of acceleration and power density. However, acceleration and power density did not vary along the different stiffness of platform A, suggesting they were unaffected by stiffness. Similarly, Astley et al. (2015) found that the power output of jumping Cuban tree frogs was not significantly affected by branch stiffness, but their energy output was.

Interestingly, although stiffness did not significantly affect acceleration and power density, there was a slight advantage of the stiffness of platform A matching the stiffness of the grasshopper, suggesting that there was an optimum platform stiffness for maximum acceleration and power density, when the mass of the platform is less than the 6.34 g platform tested here. Gilman et al. (2012) researched leaf compliance as part of their study into jumping by green anole lizards (*Anolis carolinensis*) and found that leaves had a stiffness ranging between 0.03–1.43 m N^−1^, although the species of leaf was not recorded. The measurement of leaf stiffness, which involved the application of external forces, was dependent on factors such as osmotic pressure, which varied with age of the leaf and season (Tyree et al., 1978).

The mechanical structure of leaves is formed of multiple cantilevers, i.e. a mass extending horizontally that is supported at only one fixed end (Niklas, 1999). Therefore, the interaction between the plant and an external mass (whether this be experimentally or animals jumping from it) becomes highly complex and difficult to measure. On a cellular level, the stiffness of leaves depends on the number of cells in a tissue sample, because deformation is resisted by the cell walls (Niklas, 1992). Owing to the variation of leaf stiffness and the complex relationship between a leaf and a jumper, it is difficult to suggest whether this specific platform mass and stiffness that produced an optimum acceleration and power is applicable to grasshopper locomotion in the field.

### Conclusion

It has been demonstrated that grasshoppers have the ability to recover energy from a recoiling compliant substrate, thus maintaining high velocity, acceleration and power. Biological LaMSA jumpers can successfully exploit their environment for jumping, although further exploration is necessary to conclude the mechanisms that underpin the ability of grasshoppers to do so. This research has broadened our knowledge around the incredibly energy efficient grasshopper jumping mechanisms and how they have evolved to adapt to uneven substrates.

## Supplementary Material

10.1242/jexbio.248018_sup1 Supplementary information

## References

[JEB248018C1] Astley, H. C., Haruta, A. and Roberts, T. J. (2015). Robust jumping performance and elastic energy recovery from compliant perches in tree frogs. *J. Exp. Biol.* 218, 3360-3363. 10.1242/jeb.121715 26538173

[JEB248018C2] Bailey, N. T. J. (1981). *Statistical Methods in Biology*, 2nd edn. London: Hodder & Stoughton.

[JEB248018C3] Bates, D., Mächler, M., Bolker, B. and Walker, S. (2015). Fitting linear mixed-effects models using lme4. *J. Stat. Softw.* 67, 1-48. 10.18637/jss.v067.i01

[JEB248018C4] Bennet-Clark, H. C. (1975). The energetics of the jump of the locust *Schistocerca gregaria*. *J. Exp. Biol.* 63, 53-83. 10.1242/jeb.63.1.53 1159370

[JEB248018C5] Bennet-Clark, H. C., Chapman, R. F. and Joern, A. (1990). Jumping in orthoptera. In *Biology of Grasshoppers* (ed. R. F. Chapman and A. Joern), pp. 173-203. New York: John Wiley & Sons.

[JEB248018C6] Bleichman, I., Yadav, P. and Ayali, A. (2023). Visual processing and collective motion-related decision-making in desert locusts. *Proc. R. Soc. B* 290, 20221862. 10.1098/rspb.2022.1862 PMC9845972 36651041

[JEB248018C7] Burrows, M. (2006). Jumping performance of froghopper insects. *J. Exp. Biol.* 209, 4607-4621. 10.1242/jeb.02539 17114396

[JEB248018C8] Burrows, M. and Morris, O. (2003). Jumping and kicking in bush crickets. *J. Exp. Biol.* 206, 1035-1049. 10.1242/jeb.00214 12582146

[JEB248018C9] Divi, S., Reynaga, C., Azizi, E. and Bergbreiter, S. (2023). Adapting small jumping robots to compliant environments. *J. R. Soc. Interface* 20, 20220778. 10.1098/rsif.2022.0778 36854379 PMC9974292

[JEB248018C10] Gabriel, J. M. (1985a). The development of the locust jumping mechanism: I. Allometric growth and its effect on jumping performance. *J. Exp. Biol.* 118, 313-326. 10.1242/jeb.118.1.313

[JEB248018C11] Gabriel, J. M. (1985b). The development of the locust jumping mechanism: II. Energy storage and muscle mechanics. *J. Exp. Biol.* 118, 327-340. 10.1242/jeb.118.1.327

[JEB248018C12] Gilman, C. A., Bartlett, M. D., Gillis, G. B. and Irschick, D. J. (2012). Total recoil: perch compliance alters jumping performance and kinematics in green anole lizards (*Anolis carolinensis*). *J. Exp. Biol.* 215, 220-226. 10.1242/jeb.061838 22189765

[JEB248018C13] Goode, C. K. and Sutton, G. P. (2023). Control of high-speed jumps: the rotation and energetics of the locust (*Schistocerca gregaria*). *J. Comp. Physiol. B.* 193, 145-153. 10.1007/s00360-022-01471-4 36715704 PMC9992258

[JEB248018C14] Gvirsman, O., Kosa, G. and Ayali, A. (2016). Dynamics and stability of directional jumps in the desert locust. *PeerJ* 4, e2481. 10.7717/peerj.2481 27703846 PMC5045875

[JEB248018C15] Heitler, W. J. (1977). The Locust Jump: III. Structural specializations of the metathoracis tibiae. *J. Exp Biol.* 67, 29-36. 10.1242/jeb.67.1.29

[JEB248018C16] Ilton, M., Bhamla, M. S., Ma, X., Cox, S. M., Fitchett, L. L., Kim, Y., Koh, J. S., Krishnamurthy, D., Kuo, C. Y., Temel, F. Z. et al. (2018). The principles of cascading power limits in small, fast biological and engineered systems. *Science* 360, eaao1082. 10.1126/science.aao1082 29700237

[JEB248018C17] Katz, S. L. and Gosline, J. M. (1993). Ontogenetic scaling of jump performance in the African desert locust (*Schistocerca gregaria*). *J. Exp. Biol.* 177, 81-111. 10.1242/jeb.177.1.81

[JEB248018C18] Kirkton, S. D. and Harrison, J. F. (2006). Ontogeny of locomotory behaviour in the American locust, *Schistocerca americana*: from marathoner to broad jumper. *Anim. Behav.* 71, 925-931. 10.1016/j.anbehav.2005.09.010

[JEB248018C19] Kuznetsova, A., Brockhoff, P. B. and Christensen, R. H. B. (2017). lmerTest package: tests in linear mixed effects models. *J. Stat. Softw.* 82, 1-26. 10.18637/jss.v082.i13

[JEB248018C20] Longo, S. J., Cox, S. M., Azizi, E., Ilton, M., Olberding, J. P., St Pierre, R. and Patek, S. N. (2019). Beyond power amplification: latch-mediated spring actuation is an emerging framework for the study of diverse elastic systems. *J. Exp. Biol.* 222, jeb197889. 10.1242/jeb.197889 31399509

[JEB248018C21] Mo, X., Romano, D., Miraglia, M., Ge, W. and Stefanini, C. (2020). Effect of substrates’ compliance on the jumping mechanism of *Locusta migratoria*. *Front. Bioeng. Biotechnol.* 8, 661. 10.3389/fbioe.2020.00661 32775320 PMC7381386

[JEB248018C22] Niklas, K. J. (1992). *Plant Biomechanics: An Engineering Approach to Plant Form and Function*. Chicago: University of Chicago Press.

[JEB248018C23] Niklas, K. J. (1999). A mechanical perspective on foliage leaf form and function. *New Phytol.* 143, 19-31. 10.1046/j.1469-8137.1999.00441.x

[JEB248018C25] Pond, C. M. (1972). The initiation of flight in unrestrained locusts, *Schistocerca gregaria*. *J. Comp. Physiol.* 80, 163-178. 10.1007/BF00696488

[JEB248018C26] Reynaga, C. M., Eaton, C. E., Strong, G. A. and Azizi, E. (2019). Compliant substrates disrupt elastic energy storage in jumping tree frogs. *Integr. Comp. Biol.* 59, 1535-1545. 10.1093/icb/icz069 31141102

[JEB248018C27] Scholz, M. N., Bobbert, M. F. and Van Soest, A. K. (2006). Scaling and jumping: gravity loses grip on small jumpers. *J. Theor. Biol.* 240, 554-561. 10.1016/j.jtbi.2005.10.015 16332377

[JEB248018C28] Sobel, E. C. (1990). The locust's use of motion parallax to measure distance. *J. Comp. Physiol. A.* 167, 579-588. 10.1007/BF00192653 2074547

[JEB248018C29] Sutton, G. P. and Burrows, M. (2008). The mechanics of elevation control in locust jumping. *J. Comp. Physiol. A.* 194, 557-563. 10.1007/s00359-008-0329-z 18373101

[JEB248018C30] Sutton, G. P. and Burrows, M. (2011). Biomechanics of jumping in the flea. *J. Exp. Biol.* 214, 836-847. 10.1242/jeb.052399 21307071

[JEB248018C31] Thorpe, S. K. S., Crompton, R. H. and Alexander, R. M. (2007). Orangutans use compliant branches to lower the energetic cost of locomotion. *Biol. Lett.* 3, 253-256. 10.1098/rsbl.2007.0049 17439848 PMC2464692

[JEB248018C32] Tyree, M. T., Cheung, Y. N. S., MacGregor, M. E. and Talbot, A. J. B. (1978). The characteristics of seasonal and ontogenetic changes in the tissue–water relations of *Acer*, *Populus*, *Tsuga*, and *Picea*. *Can. J. Bot.* 56, 635-647. 10.1139/b78-071

[JEB248018C33] Wallace, G. K. (1959). Visual scanning in the desert locust *Schistocerca gregaria* Forskål. *J. Exp. Biol.* 36, 512-525. 10.1242/jeb.36.3.512

[JEB248018C34] Zajac, F. E. (1989). Muscle and tendon: properties, models, scaling, and application to biomechanics and motor control. *Crit. Rev. Biomed. Eng.* 17, 359-411. 2676342

