# Supplementary material for: Kinematics and energetics of the desert locust (Schistocerca gregaria) when jumping from compliant surfaces
Source: J Exp Biol. 2024 Dec 16;227(24):jeb248018. doi: 10.1242/jeb.248018 (PMC11698038; doi:10.1242/jeb.248018)
Supplement: Supplementary information [file jexbio-227-248018-s1.pdf]

Supplementary Materials and Methods

Calculations

The numerical plot coordinates, along with a known number of frames and therefore known time (ms) between each frame, allowed for determination of various kinematics. Time to take off (s), velocity (m/s), elevation (degrees), and acceleration distance (m) values were calculated using measurements directly from the video recordings using the equations shown below. Variables, their units and symbols in equations are summarised in Table S2.

Grasshopper and platform stiffness were both required to understand the relationship between the grasshopper and the platform, which can be described by calculating the stiffness ratio between the two:

$$\boldsymbol{Stiffness\ ratio\ (\tilde{k}) = \frac{k_g}{k_p}} \qquad \textbf{Eqn S1}$$

where: k_g was grasshopper stiffness (N/m) and k_p was the platform stiffness (N/m). The greatest measured k_g from the three control jumps taken was used to calculate $\tilde{k}$ for each grasshopper at each line of each platform (described in 2.4.1).

The mass of the platform and grasshopper were used to calculate a mass ratio for the same reasons as the stiffness ratio above.

$$\boldsymbol{Mass\ ratio\ (\tilde{m}) = \frac{m_g}{m_p}} \qquad \textbf{Eqn S2}$$

Where: m_g was the mass of the grasshopper and m_p was the mass of the compliant portion of the platform. This was determined by measuring the length of the rigid portion, calculating the percentage length of the rigid portion, and subtracting this percentage from the total mass of the platform. $\tilde{m}$ was calculated for each grasshopper on each platform. More detail on the experimental platforms can be found in 2.4.1. $\tilde{k}$ and $\tilde{m}$ were calculated for a direct comparison to results report by Divi *et al.* (in review) results.

The below calculations directly relate to the various platforms' mechanical properties which are used in the experimental design (as described in 2.4.1). Platform stiffness was an independent variable throughout the experiment and were measured prior to filming (described further in 2.4.1).

$$\boldsymbol{Force\ (F) = m_p * a}$$

$$\boldsymbol{Platform\ stiffness\ (k_p) = \frac{F}{x}} \qquad \textbf{Eqn S3}$$

where: F was applied force, m_p was mass of the platform and a was acceleration (in this case this was gravitational with a value of 9.81 m/s^2), and k_p was the platform stiffness (N/m).

Acceleration distance, which is the distance between the proximal end of the femur and distal end of the tibia at maximum extension during the take-off period of the jump, was measured to calculate the grasshopper stiffness (for use in equation E11).

$$\boldsymbol{Acceleration\ distance\ (x) = 2 * L\left(\sin\frac{6.2831 * \frac{angle\ of\ leg\ extension}{2}}{360}\right)} \qquad \textbf{Eqn S4}$$

where: L = length of the grasshoppers' tibia (m) and 6.2831 was the circumference of a circle in radians. This was multiplied by ½ angle of leg extension and divided by 360 to Pythagoras'

Journal of Experimental Biology • Supplementary information

theorem was calculated in degrees.

Grasshopper stiffness of the entire metathoracic leg (i.e., a value equivalent to all spring systems found within the leg) was calculated to further understand the relationship between the grasshopper mechanics and platform mechanics. This was calculated using variables determined during the control jumps of the individual grasshoppers. The stiffness of the grasshopper must be measured from a jump from a rigid substrate and cannot be calculated from the experimental jumps. This was because spring energy will be lost to a compliant platform, and thus will have a possible effect on velocity and acceleration distance, and therefore stiffness. The derivation for this calculation was as follows:

$$\boldsymbol{Kinetic\ energy} = \frac{1}{2}\boldsymbol{m_g v^2} = \boldsymbol{elastic\ potential\ energy} = \frac{1}{2}\boldsymbol{k_g x^2}$$

$$\boldsymbol{m_g v^2 = k_g x^2}$$

$$\boldsymbol{\frac{m_g v^2}{x^2} = k_g}$$

$$\boldsymbol{Grasshopper\ stiffness\ (k_g)} = \boldsymbol{\frac{m_g v^2}{x^2}} \qquad \textbf{Eqn S5}$$

where: m_g was the grasshopper mass (kg), v was the grasshopper velocity (m/s), k_g was stiffness of the grasshopper (N/m) and x was the acceleration distance (m).

Time to take-off (ms) indicated the length of time it took for the grasshopper to reach the air (tarsi no longer in contact with the platform) from a still position (Fig. S2) which allowed the calculation of acceleration (Eqn S5), power (Eqn S8) and power density of jumping muscles (Eqn S9). Time to take-off was calculated using the following equation:

Time to take-off = last frame of tarsi-platform contact (frame number) – first frame of metathoracic leg extension (frame number) **Eqn S6**

Note: 1 frame = 1 ms at 1000 fps

Velocity (m/s) was a key variable to measure for each jump as it is widely measured and defined in literature as well as having a direct link to measuring their acceleration, kinetic energy and power. It was calculated:

$$\boldsymbol{Velocity\ (v)} = \boldsymbol{\frac{\Delta d}{0.01}} \qquad \textbf{Eqn S7}$$

where: Δd = the change in displacement (m) and 0.01 the time over which this displacement was measured (s), which was consistent throughout all velocity measurements. For example, a grasshopper could displace 0.018 m in 0.01 s, resulting in a velocity of 1.8 m/s.

Elevation in degrees (θ), was the angle at which the grasshopper jumps upwards in relation to the platform beneath them. Elevation was calculated by tracking a single point on the grasshopper across the first ten frames of movement, beginning when the tarsi left contact with the platform. Pythagoras' theorem was used to calculate theta in the following equation

$$\boldsymbol{Elevation\ (\theta)} = \left(\boldsymbol{tan^{-1}\frac{\Delta dy}{\Delta dx}}\right) * \left(\boldsymbol{\frac{180}{\pi}}\right) \qquad \textbf{Eqn S8}$$

where: Δd*y* = the displacement of the grasshopper in the y axis and Δd*x* = the displacement of the grasshopper in the x axis. The above equation could be used on its own for the control jumps, where the platform was rigid and did not move beneath the grasshopper. When jumping from a compliant platform, the platform would be displaced under the grasshopper's mass prior to any movement. Therefore, the platform displacement angle (°) though often very small, was also measured and added to theta for when measuring the total elevation of a grasshopper jump. This was measured using the same equation as theta, though the points were plotted as shown in Fig. 2.5.

$$\boldsymbol{Platform\ displacement\ angle} = \left(\boldsymbol{tan^{-1}\frac{\Delta dy}{\Delta dx}}\right) * \left(\boldsymbol{\frac{180}{\pi}}\right) \qquad \textbf{Eqn S9}$$

where: Δd*y* = the displacement of the platform in the y axis and Δd*x* = the displacement of the platform in the x axis. From here on, when referring to elevation of a grasshopper jump, theta signifies only the elevation for control jumps, and for experimental jumps theta plus the platform displacement angle. It was unknown whether grasshoppers jump at different elevations if energy was lost to a compliant platform, therefore this was investigated by

Journal of Experimental Biology • Supplementary information

measuring elevation under controlled conditions (described in 2.4.1).

These values were then used to calculate a series of other variables using the equations shown below: acceleration of the centre of mass (m/s^2), kinetic energy (J), kinetic energy density of the jumping muscles (J/kg), power (W), power density of the jumping muscles (W/kg), grasshopper stiffness (N/m) and platform stiffness (N/m).

Acceleration (m/s^2) was the velocity of the grasshopper over time and measuring this throughout all jumps allowed an investigation into whether grasshoppers maintained their high accelerations on compliant platforms (described in 2.4.1).

$$\boldsymbol{Acceleration\ (a) = \frac{v}{t}} \qquad \textbf{Eqn S10}$$

where: v = velocity (m/s) and t = time to take-off (s).

As previously outlined in Chapter 1, elastic potential energy stored within the metathoracic leg was rapidly converted into kinetic energy when released. This kinetic energy was calculated from the previously measured variables and was necessary to explore energy transfer, loss and recovery from different experimental conditions (described in 2.4.1).

$$\boldsymbol{Kinetic\ energy\ (KE) = \frac{1}{2} m_g v^2} \qquad \textbf{Eqn S11}$$

where: m_g was the mass of the grasshopper (kg) and v was the velocity of the grasshopper (m/s).

Kinetic energy density of jumping muscles, rather than kinetic energy density of the whole system, was calculated here because it allowed kinetic energy to be investigated in the context of jumping muscles only.

$$\textbf{\textit{KE density of jumping muscles (KEd)}} = \frac{\boldsymbol{KE}}{\boldsymbol{0.043 * m_g}} \qquad \textbf{Eqn S12}$$

where: KE was the kinetic energy of the whole grasshopper, and m_g was the mass of the grasshopper. The mass of jumping muscles of both metathoracic legs equates to 4.3% of body mass (m_g, in g) of a fourth instar grasshopper and 6.3% for an adult grasshopper (Gabriel, 1985). To investigate the kinetic energy density of jumping muscles in fifth instar grasshoppers, calculations were based on fourth instar jumping muscle mass, because the body mass of the animals used in this study more closely resembled the body mass of fourth instars reported by Gabriel (1985). Therefore, the mass of the whole grasshopper was multiplied by 0.043 to calculate the grasshopper's muscle mass.

Calculating power produce by the grasshopper during jumping allowed kinetic energy to be calculated with respect to time and can therefore investigate how power was affected if kinetic energy and time to take-off were also affected by the differing compliant platforms (described in 2.4.1).

$$\textbf{\textit{Power (P)}} = \frac{\boldsymbol{KE}}{\boldsymbol{t}} \qquad \textbf{Eqn S13}$$

where: KE was kinetic energy (J) and t was time to take-off (s).

Power density relative to jumping muscles was calculated for the same reason as kinetic energy density relative to jumping muscles. This allowed changes in power density to be investigated in the context of jumping muscles only rather than looking at the whole system. This described the effect of platform on power density while exploring power produced by the metathoracic legs in isolation.

$$\textbf{\textit{Power density relative to jumping muscles (Pd)}} = \frac{\boldsymbol{KE}}{\boldsymbol{0.043 * m_g * t}} \qquad \textbf{Eqn S14}$$

where: KE was kinetic energy of the grasshopper (J), m_g was the mass of the grasshopper (kg) and t was the time to take-off (s). The mass used in the equation was the mass of the jumping muscles only, which was 4.3% of their body mass, which was why it is multiplied by 0.043 in this equation.

Statistical analysis

$$\boldsymbol{Variable\ proportion\ (\widetilde{variable}) = \frac{(variable)_g}{(variable)_c}} \qquad \textbf{Eqn S15}$$

where: $(\text{variable})_g$ was the grasshoppers' experimental value for a given calculated variable

Journal of Experimental Biology • Supplementary information

and $(variable)_c$ was the mean of the same grasshoppers' control values for the same calculated variable.

$$\boldsymbol{Elevation\ difference = \ elevation_g - \ mean\ of\ elevation_c} \qquad \textbf{Eqn S16}$$

where: $elevation_g$ was the grasshoppers' experimental calculated elevation and $elevation_c$ was the mean of the same grasshoppers' control elevation.

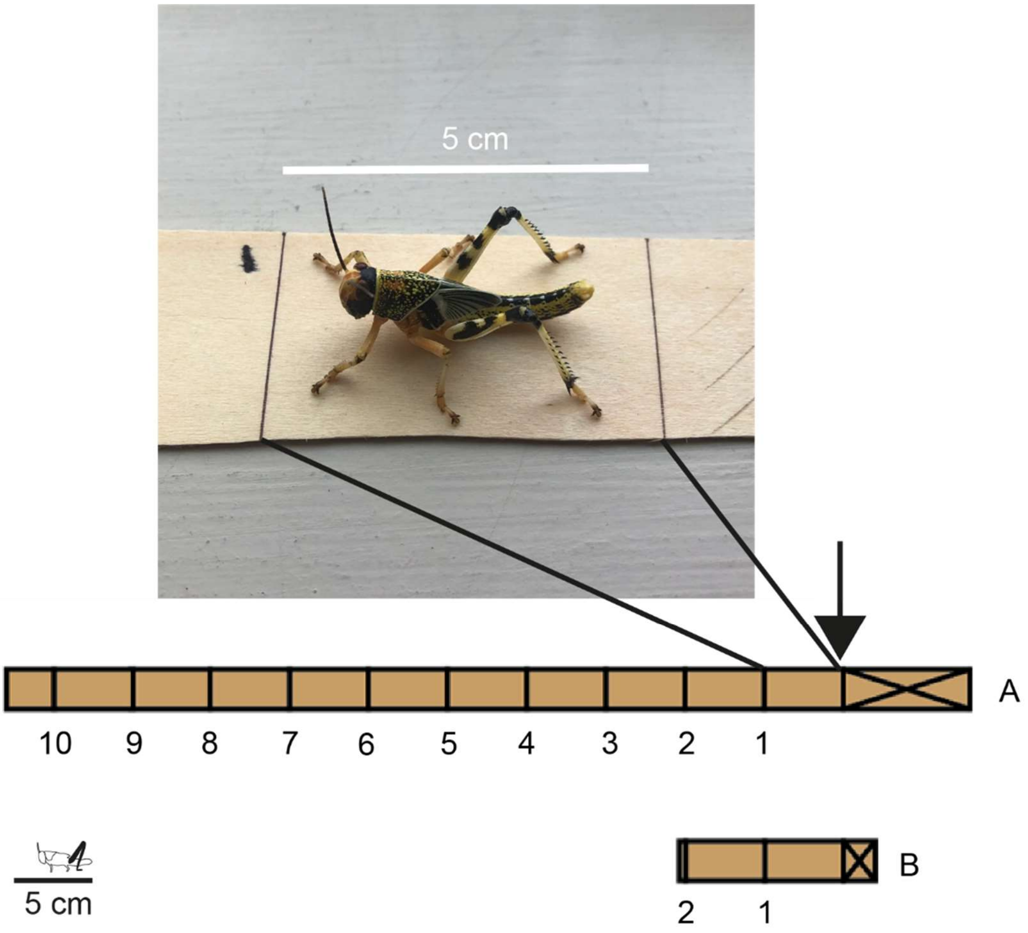

Fig. S1. Above: a fifth instar grasshopper photographed from above between lines 8 and 9 on platform A. Below: platform A and B illustrated to scale with marked lines from 1 (most stiff) to 10 (least stiff). The 'X' marked portions are fixed to a rigid, elevated wooden box beneath. An illustrated grasshopper in the bottom left to visualise the scale. The arrow indicates the point at which the first measurement of 5cm increments was taken, measuring from right to left on each platform.

Journal of Experimental Biology • Supplementary information

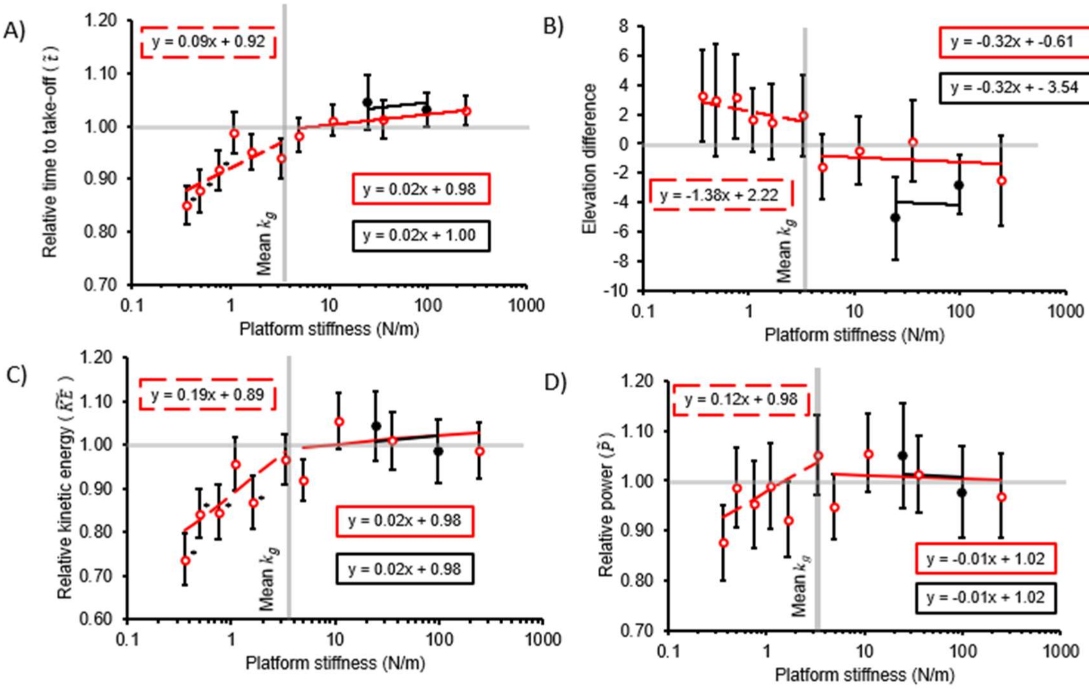

Fig. S2. Mean (± SE) values for other variables across platform A and B and different platforms. A) Relative time to take-off. B) Elevation difference. C) Relative kinetic energy. D) Relative power. The horizontal grey lines mark a decrease in the variable compared to the control jumps when below it. The vertical grey bars show the mean grasshopper stiffness. An asterix indicates data points significantly differing from 1.0 (where t_g = mean t_c). Trendlines were generated based on output from linear mixed effect regression analysis of the data (see Table 2).

Journal of Experimental Biology • Supplementary information

Table S1. Details of the trial platform's dimensions measured stiffnesses (N/m) at each line. and the number of experimental animals for each of the control and trial recordings together with the total number of jumps for three different trial platforms (see Section 2.4 for more details). N/A = not applicable.

Line	Platform A	Platform B
Thickness (cm)	0.2	0.2
Width (cm)	2.5	2.5
Length (cm)	61	12.4
Total mass (g)	6.34	1.01
Fixed platform (%)	13	16
Compliant mass (g)	5.51	0.85
Stiffness at line 1 (N/m)	245	98
Stiffness at line 2 (N/m)	35.64	24.5
Stiffness at line 3 (N/m)	10.89	N/A
Stiffness at line 4 (N/m)	4.9	N/A
Stiffness at line 5 (N/m)	3.27	N/A
Stiffness at line 6 (N/m)	1.63	N/A
Stiffness at line 7 (N/m)	1.09	N/A
Stiffness at line 8 (N/m)	0.7	N/A
Stiffness at line 9 (N/m)	0.49	N/A
Stiffness at line 10 (N/m)	0.36	N/A
No. control animals	21	19
No. control jumps	49	40
No. experimental animals	21	19
No. experimental jumps	188	31

Journal of Experimental Biology • Supplementary information

Table S2. Summary of variables and their descriptions used in the following equations. In the table and the text, a variable with a subscript of "$(variable)_c$" refers to a variable of a control grasshopper jump, a subscript of "$(variable)_g$" refers to a variable of an experimental grasshopper jump, and a subscript of "$(variable)_p$" refers to a variable of the platform.

Description	Variable symbol	Units
Velocity	v	m/s
Mass of grasshopper	m_g	g
Mass of platform	m_p	g
Mass ratio between grasshopper and platform	$\tilde{m}$	N/A
Acceleration distance	x	m
Acceleration	a	m/s^2
Displacement	d	m
Displacement (y axis only)	dy	m
Displacement (x axis only)	dx	m
Length of tibia	L	m
Time to take-off	t	ms
Stiffness of grasshopper	k_g	N/m
Stiffness of platform	k_p	N/m
Stiffness ratio between grasshopper and platform	$\tilde{k}$	N/A
Grasshopper elevation	θ	°
Kinetic energy	KE	mJ
Kinetic energy density relative to jumping muscles	KEd	J/kg
Power	P	mW
Power density relative to jumping muscles	Pd	W/kg

Journal of Experimental Biology • Supplementary information

Table S3. Mean (± SE) values for variables from control jumps from testing trials involving Platform A and B together with the results of linear mixed-effect modelling using Satterthwaite's method to test for the effect of trial (ANOVA). Sample sizes (n) per platform are indicate in the first column. In all cases, no significant effects of testing batch were found and so data were combined as indicated in the last row.

Variable	Mass (m; g)	Grasshopper stiffness (k; N/m)	Time to take-off (t; s)	Velocity (v; m/s)	Elevation (°; degrees)	Acceleration (a; m/s^2)	Kinetic energy (KE; mJ)	Kinetic energy density (KEd; J/kg)	Power (P; mW)	Power density (Pd; W/kg)
Platform A (n = 21)	1.18 ± 0.06	3.20 ± 0.15	0.034 ± 0.001	1.42 ± 0.04	41.24 ± 1.60	43.91 ± 1.90	1.18 ± 0.067	24.45 ± 1.40	36.69 ± 2.26	767.51 ± 50.63
Platform B (n = 19)	1.07 ± 0.03	3.63 ± 0.14	0.031 ± 0.001	1.46 ± 0.04	46.27 ± 1.30	48.77 ± 1.80	1.14 ± 0.054	25.20 ± 1.19	38.36 ± 2.18	848.96 ± 48.50
ANOVA F_{DF} (p-value)	$F_{1.38}$ = 2.51 (0.12)	$F_{1,38.59}$ = 2.42 (0.13)	$F_{1,39.02}$ = 6.13 (0.02)	$F_{1,39.24}$ = 0.07 (0.79)	$F_{1,36.34}$ = 4.39 (0.04)	$F_{1,38.82}$ = 1.73 (0.20)	$F_{1,38.99}$ = 0.32 (0.57)	$F_{1.3921}$ = 0.01 (0.92)	$F_{1,38.75}$ = 0.08 (0.78)	$F_{1,39.11}$ = 0.57 (0.45)
Combined values (n = 40)	1.13 ± 0.04	3.39 ± 0.11	0.032 ± 0.0005	1.44 ± 0.03	43.50 ± 1.08	46.09 ± 1.34	1.17 ± 0.044	24.79 ± 0.93	37.44 ± 1.58	804.12 ± 35.45

Table S4. Results of linear mixed effects models comparing values for various variables relative to control jumps from trials involving Platform A and B together with the results of linear mixed-effect modelling using Satterthwaite's method to test for the effect of platform stiffness and platform type, whilst controlling for animal as a random factor. Where no significant interactions were observed (see text) the simplified models were reported.

Variable	Kinetic energy ($\widetilde{KE}$; J/kg)	Power ($\tilde{P}$; W/kg)
**Platform stiffness < grasshopper stiffness**		
Slope	0.1893	0.1151
Intercept A	0.8863	0.9783
Platform	$F_{1,90.476}$ = 19.2080 (<0.00001)	$F_{1,90.72}$ = 3.8347 (0.0533)
**Platform stiffness > grasshopper stiffness**		
Slope	0.0199	-0.0070
Intercept A	0.9813	1.0173
Intercept B	0.9801	1.0224
Log Platform Stiffness	$F_{1,68.871}$ = 0.3081 (0.581)	$F_{1,71.143}$ = 0.0232 (0.879)
Platform	$F_{1,41.953}$ = 0.0003 (0.987)	$F_{1,44.686}$ = 0.0029 (0.958)

Journal of Experimental Biology • Supplementary information
